# Identification of the *ZmDUF966* Gene Family in Maize, Analysis of Its Expression Under Cold Stress, and Preliminary Investigation of the *ZmDUF966-10* Regulatory Network

**DOI:** 10.3390/biology15060514

**Published:** 2026-03-23

**Authors:** Minghao Sun, Wenyue Li, Yunlong Li, Sinan Li, Yan Sun, Shujun Li, Yue Yin, Enhao Zhou, Yue Wang, Tao Yu, Wei Zhao, Quan Cai, Xin Li, Jianguo Zhang

**Affiliations:** 1 Postdoctoral Research Station, Heilongjiang Academy of Agricultural Sciences, Harbin 150086, China; sunminghao_yg@yeah.net (M.S.); yutaoweiwei@163.com (T.Y.); 2 Heilongjiang Academy of Agricultural Sciences, Harbin 150086, China; 3 Key Laboratory of Biology and Genetics Improvement of Maize in Northern Northeast Region, Ministry of Agriculture and Rural Affairs, Harbin 150086, China

**Keywords:** *maize*, *ZmDUF966*, cold stress, miRNA regulation, ABA/WDS protein

## Abstract

Maize (*Zea mays* L.) originated in tropical and subtropical regions. During its growth and development, cold stress severely threatens seedling survival rates and final yield by inducing oxidative stress, compromising cell membrane integrity, and causing “physiological drought.” The Domain of Unknown Function 966 (*DUF966*) gene family comprises a class of regulatory factors containing conserved domains of undetermined function. Although they are considered to be extensively involved in plant growth, development, and stress response, their specific roles within the maize cold-tolerance regulatory network remain to be explored. In this study, 10 *ZmDUF966* family members were identified via genome-wide analysis, and their phylogenetic relationships, gene structures, conserved motifs, chromosomal localizations, and cis-acting elements were systematically analyzed. The results indicate that the *ZmDUF966* family is highly conserved among Poaceae species, and its promoters are enriched with stress-responsive elements such as LTR and ABRE. The core gene, *ZmDUF966-10*, was significantly up-regulated (approximately 35-fold at 48 h, *p* < 0.05) as validated by RT-qPCR under cold stress and is post-transcriptionally regulated by conserved miRNAs such as zma-miR159. Further yeast two-hybrid experiments revealed a preliminary physical interaction between the ZmDUF966-10 protein and an ABA/WDS-induced protein, suggesting its potential involvement in ABA-mediated stress signaling, though functional validation remains to be conducted. In conclusion, this study identifies *ZmDUF966-10* as a promising candidate gene that responds to cold signals through multi-level regulatory networks, providing a valuable gene resource for further functional characterization and potential application in cold-tolerant maize improvement.

## Simple Summary

Cold stress significantly limits maize growth and yield, particularly at the seedling stage. In this study, we systematically identified and characterized 10 members of the maize *DUF966* gene family and analyzed their potential roles in cold stress response. Among them, *ZmDUF966-10* was strongly induced under low-temperature conditions and may be regulated by conserved microRNAs and ABA-related signaling pathways. Preliminary interaction analysis suggests that this gene participates in stress-responsive regulatory networks. These findings provide a promising candidate gene for future functional studies and for improving cold tolerance in maize breeding programs.

## 1. Introduction

Maize (*Zea mays* L.) is a tropical crop domesticated from its wild ancestor, teosinte (*Z. mays* subsp. *parviglumis*), with an optimal growth temperature of 25–28 °C. Abiotic stresses such as drought, salinity–alkalinity, and low temperature not only affect the quality of maize seedlings but also severely limit the yield and quality of maize [1,2,3]. Because cold stress reduces the germination rate of seeds and decreases seedling vigor, low temperatures cause irreversible damage to the cells of maize seedlings, leading to membrane damage, loss of cellular components, and permanent alterations in chemical properties [4]. When maize seedlings are subjected to cold stress, plant height, root length, chlorophyll content, and net photosynthetic rate decrease, directly resulting in growth retardation and phenomena such as leaf wilting, necrosis, or even death [5]. Furthermore, seedlings subjected to cold stress are highly susceptible to infection by soil-borne bacteria [6].

Domain of Unknown Function (DUF) is a recognized conserved region within a protein structure, designated as Pfam ID PF06136. It collectively refers to domains within the Pfam database that have not yet been experimentally validated. These domains exhibit two defining characteristics: they are composed of relatively conserved amino acid sequences, and their biological functions remain unidentified [7]. Proteins containing DUF domains are classified into different families based on specific conserved domains. Numerous studies have demonstrated that DUF family genes play critical roles in plant growth and development across various species [8]. For example, in *Arabidopsis*, the *DUF724* family regulates polar cell growth in apical meristems [9]; DUF579 family members are essential for the 4-O-methylation of glucuronic acid in cell wall components [10]. In rice (*Oryza sativa*), *OsRMC* of the *DUF26* family negatively regulates root development [11], while *DUF26* family members in other species positively regulate responses to fungal and bacterial pathogens [12,13]. Overexpression of *DUF966* genes in rice can enhance plant tolerance to salt and PEG-simulated drought stress [14]. Additionally, *StDDP5* and *StDDP7*, belonging to the *DUF221* gene family, exhibited high expression in response to heat and salt stress, respectively, in potato (*Solanum tuberosum*) [15]. Analysis of the DUF221 domain (DDP) in tomato (*Solanum lycopersicum*) revealed that *SlDDP1*, *SlDDP2*, *SlDDP3*, *SlDDP4*, *SlDDP7*, *SlDDP8*, and *SlDDP10* are induced under salt, drought, ABA, and IAA stress [16].

The *DUF966* gene family is widely distributed in monocots, dicots, and bryophytes, typically containing one or two highly conserved DUF966 domains [17]. Currently, the molecular functions of *DUF966* family members are not fully understood, but some studies have found their involvement in plant responses to abiotic stress. In tomato, *JWS19* is a member of the *DUF966* family that can be rapidly induced by salt stress [18]. In rice, overexpression of *OsDSR2* (a *DUF966* family member) enhances plant sensitivity to salt and drought and weakens its response to ABA [14]. Conversely, in *Arabidopsis*, *AtST39* positively regulates plant tolerance to salt and drought stress. Recently, 28 *TaDUF966* genes were identified in wheat, and VIGS experiments confirmed that *TaDUF966-9B* is involved in wheat salt tolerance [18]. These findings indicate that the *DUF966* gene family possesses potential functions in plant stress responses, warranting further investigation.

Despite the significant roles played by *DUF966* genes in plants, the *ZmDUF966* gene family in maize remains largely uncharacterized. To address this knowledge gap, the present study firstly aimed to perform genome-wide identification and systematic characterization of the *ZmDUF966* gene family, including analysis of physicochemical properties, gene structures, evolutionary relationships, and promoter cis-acting elements; Secondly, analyze the expression patterns of *ZmDUF966* family members under cold stress using transcriptomic data and RT-qPCR validation; and lastly conduct a preliminary investigation of the ZmDUF966-10 regulatory network through miRNA prediction and yeast two-hybrid protein interaction screening. These findings are expected to provide a theoretical foundation for further elucidating the molecular mechanisms of cold resistance in maize.

## 2. Materials and Methods

### 2.1. Plant Materials and Treatment

Maize (*Zea mays* L., genotype B73) seeds were maintained and propagated by our laboratory. Seeds were imbibed in deionized water for 24 h and subsequently sown into plastic pots containing a mixture of vermiculite and soil (1:1, *v*/*v*). The seedlings were cultivated in a controlled-environment growth chamber under a 16 h light/8 h dark photoperiod, with temperatures maintained at 25 °C during the day and 22 °C at night. Upon reaching the one-month-old stage (V3 stage, three-leaf-one-heart), the seedlings were subjected to cold stress at 4 °C under the same photoperiod conditions (16 h light/8 h dark). Leaf tissues were harvested at 0, 1, 3, 6, 12, 24, 36, and 48 h post-treatment. For each time point, approximately 0.1 g of tissue was collected from three biological replicates, with each sample subjected to three technical replicates. All harvested tissues were immediately snap-frozen in liquid nitrogen and stored at −80 °C for further analysis.

### 2.2. ZmDUF966 Gene Family Identification and Physicochemical Analysis

The maize genomic dataset (Zm-B73-REFERENCE-NAM-5.0), including protein sequences and annotation files, was retrieved from Phytozome (https://phytozome-next.jgi.doe.gov/, accessed on 23 November 2025).

To identify potential *DUF966* members, HMMER v3.3 was employed to search against the maize proteome using the DUF966-specific Hidden Markov Model (HMM, PF06136) with an E-value threshold of 1 × 10^5^ [19]. Meanwhile, a BLASTP (https://blast.ncbi.nlm.nih.gov/Blast.cgi?PROGRAM=blastp&PAGE_TYPE=BlastSearch&LINK_LOC=blasthome/, accessed on 23 November 2025) search was performed using known DUF966 protein sequences from *Arabidopsis thaliana* and *Oryza sativa* as query sequences to compare against maize protein sequences, thereby identifying candidate genes. After merging the candidate gene IDs, the presence of the core DUF966 domain was further validated using the SMART (http://smart.embl.de/, accessed on 23 November 2025), NCBI Conserved Domain Database (NCBI-CDD) (https://www.ncbi.nlm.nih.gov/Structure/cdd/wrpsb.cgi?SEQUENCE/, accessed on 23 November 2025), and Pfam platforms (https://pfam.xfam.org/, accessed on 23 November 2025) [20]. Sequences lacking the characteristic DUF966 domain were manually excluded. The physicochemical properties of the confirmed ZmDUF966 proteins were analyzed via ProtParam (https://web.expasy.org/protparam/, accessed on 23 November 2025), and their subcellular localizations were predicted using DeepLoc-2.0 (https://services.healthtech.dtu.dk/services/DeepLoc-2.0/, accessed on 23 November 2025).

### 2.3. Phylogenetic Analysis

The Neighbor-Joining (NJ) method was selected for its computational efficiency and its widespread use in gene family studies of similar scale. To elucidate the evolutionary relationships among *DUF966* gene family members in maize and different species. A phylogenetic tree comprising DUF966 proteins from maize, wheat (*Triticum aestivum*), rice, and *Arabidopsis* was constructed using the Neighbor-Joining (NJ) method in MEGAX [21]. Multiple sequence alignment was conducted using Clustal W v2.1. The Poisson model was adopted for evolutionary distance calculations, with 1000 bootstrap replicates to assess node reliability. The resulting tree was visualized and refined using Evolview3 (https://www.evolgenius.info/evolview/, accessed on 24 November 2025). The Ka/Ks values of *ZmDUF966* gene family members were then calculated using the ‘Simple Ka/Ks Calculator (NG)’ module in TBtools v2.376 software.

### 2.4. Chromosomal Localization and Synteny Analysis

The physical distribution of *ZmDUF966* genes across the 10 maize chromosomes was visualized using TBtools based on the genomic coordinates provided in the annotation files [22]. Intraspecific synteny within the maize genome and interspecific collinearity among maize, rice, wheat, and barley (*Hordeum vulgare*) were analyzed and mapped utilizing TBtools.

### 2.5. ZmDUF966 Family Conserved Domain and Gene Structure Analysis

Conserved motifs of ZmDUF966 proteins were identified using MEME V5.5.7 (http://meme-suite.org/, accessed on 30 November 2025) with the following parameters: a minimum motif width of 6, a maximum width of 100, and a maximum of 15 motifs. Additionally, the exon-intron structures of the *ZmDUF966* genes were extracted from the maize genome annotation files and visualized to characterize their structural diversity [23].

### 2.6. Cis-Acting Element Analysis

To identify putative *cis*-acting regulatory elements, the 2.0 kb sequences upstream of the start codon (ATG) for all *ZmDUF966* genes were extracted from the maize genome. These promoter sequences were submitted to the PlantCARE (https://bioinformatics.psb.ugent.be/webtools/plantcare/html/, accessed on 30 November 2025) database for functional annotation [24]. The identified elements were subsequently visualized using clusterProfiler R package (v4.0) following the methodological framework described by Zhang et al. [25].

### 2.7. miRNA Analysis of ZmDUF966 Gene Family Members

The full-length CDS of the ten *ZmDUF966* genes were utilized as candidate targets. Target prediction was executed via the psRNATarget server (https://www.zhaolab.org/psRNATarget/, accessed on 30 November 2025) (Expectation ≤ 5.0; UPE ≤ 25), an expectation value of ≤5.0 was applied to ensure comprehensive prediction coverage. The regulatory network of miRNA-mRNA interactions was subsequently constructed and visualized using Cytoscape software v1.10.3 [26].

### 2.8. GO Annotation and Enrichment Analysis

Functional annotation of the maize proteome-wide sequences was performed using the EggNOG-mapper online tool (http://eggnog5.embl.de/#/app/home/, accessed on 30 November 2025) to retrieve Gene Ontology (GO) annotations for *ZmDUF966* family members. Subsequently, the R package clusterProfiler (v4.0) was employed to conduct functional enrichment analysis on the identified *ZmDUF966* genes using the whole maize genome (Zm-B73-REFERENCE-NAM-5.0) as the background gene set, encompassing three categories: Biological Process (BP), Cellular Component (CC), and Molecular Function (MF) [27]. Adjusted *p*-values were calculated using the Benjamini–Hochberg method for false discovery rate (FDR) correction, with a significance threshold of adjusted *p* < 0.05. Finally, the enrichment terms associated with stress resistance were visualized utilizing TBtools or the ggplot2 plotting package in R.

### 2.9. Expression Analysis of ZmDUF966 Genes Based on Stress Transcriptome Data

Cold stress-related transcriptomic datasets (Accession No. PRJNA244661) were retrieved from the NCBI SRA database (https://static.pubmed.gov/portal/portal.fcgi/, accessed on 30 November 2025). This datasets was generated from maize (B73) seedlings subjected to cold treatment (4 °C), with control and cold-stressed samples harvested at matched time points. Gene expression quantification was performed using Salmon software v3.10.4 [28], from which the Transcripts Per Million (TPM) values for *ZmDUF966* family members were extracted. Subsequently, expression heatmaps were generated utilizing TBtools [29].

### 2.10. RT-qPCR and Data Analysis

Total RNA from leaf tissues was extracted using the TransZol UP kit (Vazyme, Nanjing, China) and subsequently reverse-transcribed into cDNA. Gene-specific primers were designed via NCBI Primer-BLAST (https://www.ncbi.nlm.nih.gov/tools/primer-blast/index.cgi?LINK%20LOC=BlastHome/, accessed on 30 November 2025) (Table S1), with *ZmActin* serving as the internal reference gene for normalization [30]. Real-time quantitative PCR (RT-qPCR) was performed on a QuantStudio 3 system using the TransScript TOP Green qPCR SuperMix (TransGen Biotech, Beijing, China). The relative expression levels were calculated utilizing the 2^−ΔΔCt^ method [31]. Data are presented as mean ± standard error of three biological replicates, each with three technical replicates. Statistical significance was determined by one-way analysis of variance (ANOVA) followed by Duncan’s multiple range test (*p* < 0.05) using SPSS software (version IBM SPSS Statistics 27). Graphical representations were generated in GraphPad Prism v10.6.0.

### 2.11. Yeast Library Screening

Bait Construction and Auto-activation Assay: The full-length CDS of ZmDUF966-10 was cloned into the pGBKT7 vector. The bait plasmid and the empty pGADT7 vector were co-transformed into Y2H yeast competent cells using the PEG/LiAc method. Transformants were subsequently inoculated onto DDO (SD/-Trp/-Leu), TDO (SD/-Trp/-Leu/-His), and QDO (SD/-Trp/-Leu/-His/-Ade) media (Coolaber, Beijing, China). The auto-activation activity and potential cytotoxicity of the bait protein were evaluated by monitoring colony growth.

Library Screening and Validation: The pGBKT7-*ZmDUF966-10* bait strain was co-transformed with a maize cDNA library constructed from leaf tissues under cold stress conditions (4 °C, 48 h), previously generated and stored in our laboratory, and plated on TDO medium for primary screening. The total number of clones (CFU) was calculated to assess library coverage. Primary positive clones were then transferred onto QDO high-stringency selective medium supplemented with X-α-Gal (Coolaber, Beijing, China) to identify stable interacting clones characterized by normal growth and blue coloration.

Clone Identification and Sequencing: PCR amplification was performed on candidate positive monocultures using specific primers (T7/AD-R). Products exhibiting distinct, single bands were recovered and sequenced. The obtained sequences were subjected to BLAST analysis via NCBI to identify potential interacting proteins of ZmDUF966-10.

### 2.12. Yeast Two-Hybrid (Y2H) Assay

The Y2H assay was performed according to the instructions in ‘Matchmaker Gold Yeast Two-Hybrid System User Manual’ (Clontech, Mountain View, CA, USA). The coding sequence of *ZmDUF966-10* was amplified by PCR and cloned into pGBKT7 vector as the bait to avoid automatic activation, and the full-length CDS of Zm00001eb362480 (ABA/WDS induced protein), Zm00001eb285440 (PROTEASE DO-LIKE 9) and Zm00001eb409220 (photosystem I subunit PsaN) was cloned into pGADT7 vector as the prey. Each prey construct was co-transformed with pGBKT7-*ZmDUF966-10* into the Y2H Gold yeast strain. At the same time, the combination of pGBKT7-53 + pGADT7-T and pGBKT7+ pGADT7 were co-transformed into Y2H Gold yeast strain as positive and negative controls, respectively. The yeast cells were cultured on SD/-Leu/-Trp, SD/-Leu/-Trp/-His and SD/-Leu/-Trp/-His/-Ade selective medium, and the interaction was evaluated.

## 3. Results

### 3.1. Identification and Physicochemical Properties of ZmDUF966 Family Members

**Figure 1:**
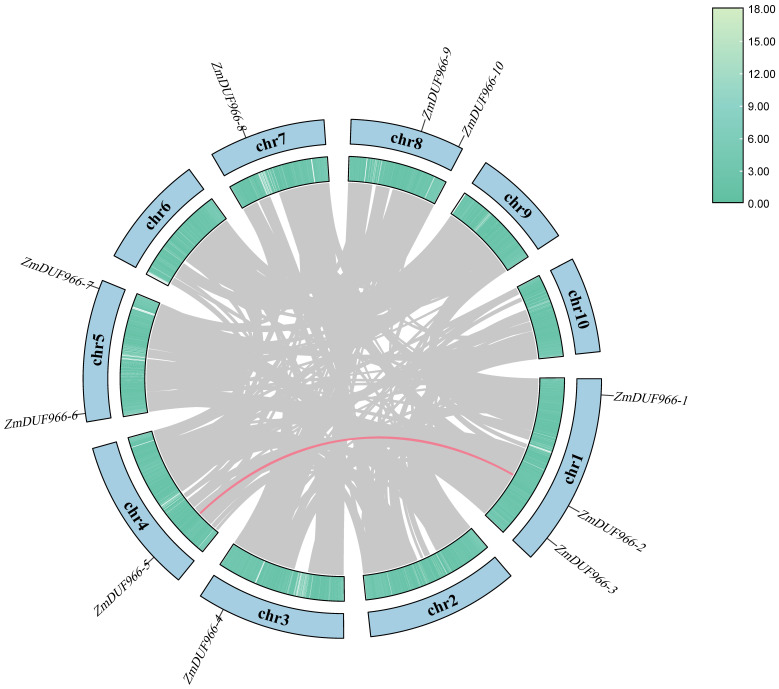
Chromosomal localization and synteny analysis of the *ZmDUF966* gene family in maize. The colored blocks representing chr-chr10 illustrate the physical distribution of the 10 *ZmDUF966* genes across the 10 maize chromosomes. In the inner circle, gray lines represent collinear blocks across the entire maize genome, while red lines specifically indicate segmental duplication events among the family members on the chromosomes.

Through genome-wide identification, 10 members containing the conserved DUF966 domain were identified in the maize (*Zea mays* L.) genome and designated as *ZmDUF966-1* to *ZmDUF966-10* according to their chromosomal positions. Visualization of the chromosomal distribution of these ten *ZmDUF966* genes (Figure 1) revealed that they are non-randomly distributed across eight maize chromosomes (Chr1–Chr8), whereas no members were identified on Chr9 or Chr10. Specifically, Chr1 and Chr2 each harbor two members, while the remaining chromosomes each contain a single member. Circos analysis identified significant collinear gene pairs within the family (e.g., *ZmDUF966-2/5*), suggesting that whole-genome duplication (WGD) or segmental duplication served as the primary drivers for the expansion of the *ZmDUF966* family in maize.

To further elucidate the biological characteristics of this protein family, a systematic analysis of their fundamental physicochemical properties was conducted (Table S2). The results demonstrated a substantial variation in protein length among *ZmDUF966* family members, ranging from 322 amino acids (aa) (ZmDUF966-10) to 635 aa (ZmDUF966-6), with molecular weights (MW) distributed between 35,622.61 Da and 70,253.23 Da. The isoelectric points (pI) of these family members ranged from 7.24 (ZmDUF966-8) to 9.78 (ZmDUF966-10); notably, the pI of all members exceeded 7.0, indicating that ZmDUF966 proteins are predominantly basic. Grand average of hydropathy (GRAVY) analysis revealed that all ZmDUF966 proteins possessed negative GRAVY values (ranging from −1.17 to −0.56), characterizing them as hydrophilic proteins. Regarding subcellular localization predictions, the ZmDUF966 family exhibited distinct spatial distribution patterns: six members (ZmDUF966-2/3/5/6/7/10) were localized to the cytoplasm, while the remaining four were localized to the nucleus. This diversity in localization suggests that family members may perform divergent regulatory functions within maize cells. It should be noted that these subcellular localizations were inferred solely from computational predictions using DeepLoc-2.0 and may not fully reflect actual in planta localization.

### 3.2. Phylogenetic and Interspecific Syntenic Analysis of the ZmDUF966 Family

**Figure 2:**
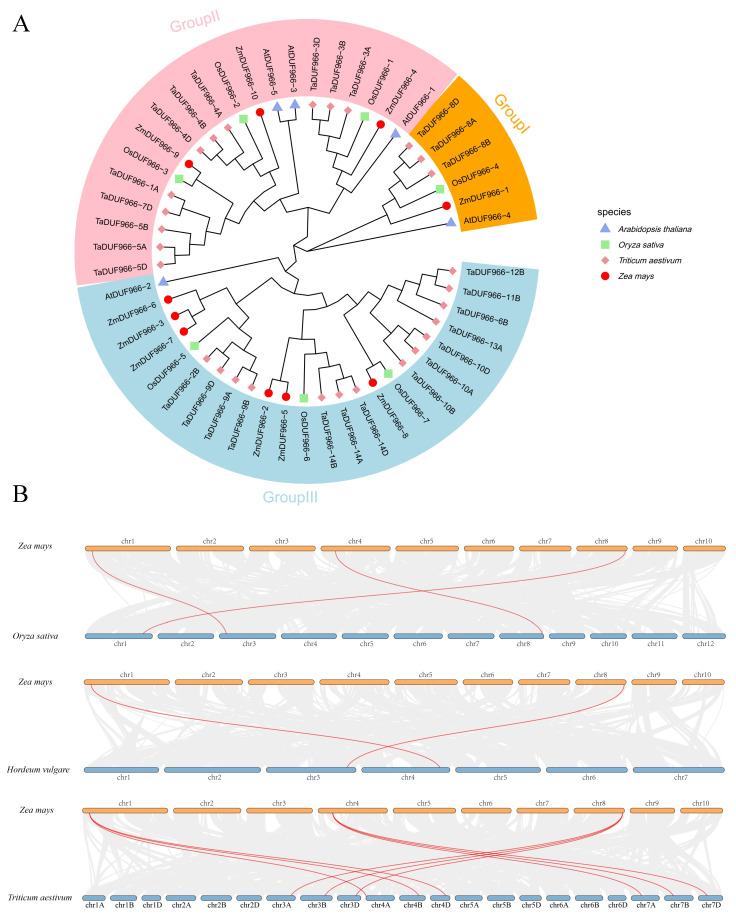
Phylogenetic analysis and interspecific synteny of the ZmDUF966 family. (**A**) Phylogenetic tree of DUF966 proteins from *Arabidopsis*, rice, wheat, and maize. Branches of different colors represent distinct subfamilies (Groups I, II, and III). Geometric symbols of different shapes and colors denote different species: red circles for maize (*Zea mays*), green squares for rice (*Oryza sativa*), pink stars for wheat (*Triticum aestivum*), and purple triangles for *Arabidopsis thaliana*. (**B**) Syntenic analysis of *DUF966* family members between maize and three other poaceous species (rice, barley, and wheat). Gray lines indicate syntenic blocks between the maize genome and other species, while red lines highlight the orthologous *DUF966* gene pairs across species. The comparisons, from top to bottom, show maize vs. rice (*Oryza sativa*), maize vs. barley (*Hordeum vulgare*), and maize vs. wheat (*Triticum aestivum*).

To investigate the phylogenetic relationships and evolutionary history of the *ZmDUF966* family, a Neighbor-Joining (NJ) phylogenetic tree was constructed using DUF966 protein sequences from *Arabidopsis thaliana*, *Oryza sativa*, *Triticum aestivum*, and *Zea mays* (Figure 2A). The results demonstrated that the DUF966 members from these four species were distinctly categorized into three major subfamilies: Group I, Group II, and Group III. The 10 *ZmDUF966* members were distributed across all three subfamilies, exhibiting clear evolutionary divergence: Group I contains *ZmDUF966-1* and *ZmDUF966-8*; Group II includes *ZmDUF966-4*, *ZmDUF966-9*, and *ZmDUF966-10* (the primary focus of this study); and Group III is the most populous subfamily, encompassing *ZmDUF966-2/3/5/6/7*. Notably, maize members exhibited shorter evolutionary distances and tended to cluster within the same clades as other monocotyledonous species (rice and wheat), while remaining phylogenetically distant from the dicotyledonous *Arabidopsis*, consistent with the established evolutionary patterns of monocot-dicot divergence.

To further elucidate the evolutionary mechanisms and orthologous relationships of the *ZmDUF966* gene family, interspecific syntenic maps were constructed between maize and three representative poaceous crops: rice, barley (*Hordeum vulgare*), and wheat (Figure 2B). The synteny analysis revealed extensive orthologous relationships between *ZmDUF966* family members and those in other poaceous species. Specifically, multiple orthologous gene pairs were identified between maize and rice, reflecting high evolutionary conservation between the two species. Syntenic associations remained significant in comparisons with barley and wheat, with the analysis of hexaploid wheat revealing more complex orthologous matching patterns. These results suggest that the *ZmDUF966* gene family has maintained remarkable evolutionary stability in terms of genomic localization and functional sequences since their divergence from a common ancestor of the Poaceae family.

To further characterize the evolutionary forces acting on the *ZmDUF966* family, the Ka/Ks ratios for homologous gene pairs were calculated (Table S3). The results showed that the Ka/Ks ratios for all identified pairs were significantly less than 1.0, ranging from 0.150 to 0.414. Notably, the *ZmDUF966-2/ZmDUF966-5* pair exhibited the lowest Ka/Ks value (0.150). These findings strongly suggest that the *ZmDUF966* family in maize has undergone intense purifying selection throughout its long evolutionary history, indicating that the family members have maintained high sequence conservation and that their biological functions remained stable during maize speciation.

### 3.3. Gene Structure and Conserved Motif Analysis of the ZmDUF966 Family

**Figure 3:**
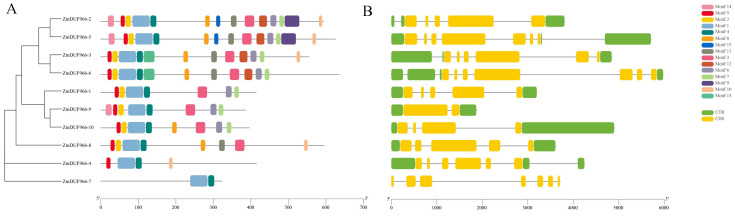
Phylogenetic relationships, conserved motifs, and gene structure analysis of the *ZmDUF966* family in maize. (**A**) Phylogenetic relationship and conserved motif analysis. The left panel shows the phylogenetic tree constructed based on full-length ZmDUF966 protein sequences; the right panel displays the predicted conserved motifs. Boxes of different colors represent different motifs (Motif 1–15), and their arrangement illustrates the distribution patterns of conserved motifs among family members. (**B**) Gene structure analysis. The diagram illustrates the genomic composition of *ZmDUF966* family members. Yellow boxes represent the CDS (coding sequences), green boxes represent UTRs (untranslated regions), and black lines represent introns.

Conserved motifs within ZmDUF966 protein sequences were identified using MEME software v5.5.9, with a total of 15 motifs (Motif 1–15) recognized (Figure 3A). The results indicated that members belonging to the same subfamily shared highly similar motif compositions. For instance, Group III members (e.g., ZmDUF966-2/5/3/6) harbored a complete set of Motifs 1–15, demonstrating remarkable sequence conservation. In contrast, the key member ZmDUF966-10 in Group II lacked Motifs 11, 13, and 15, which might be associated with its functional specialization. Gene structure analysis (Figure 3B) revealed that the number of exons in ZmDUF966 family members varied from 2 to 10. Despite differences in exon lengths, members within the same clade exhibited high consistency in their intron-exon arrangement patterns. This structural conservation aligns with the distribution of conserved motifs, further supporting the reliability of the phylogenetic classification.

### 3.4. Cis-Acting Element Analysis of ZmDUF966 Gene Promoters

**Figure 4:**
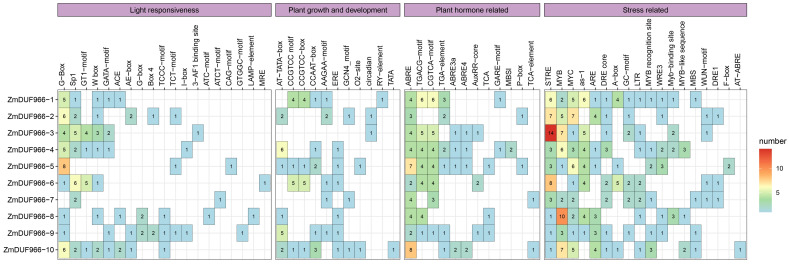
Distribution of cis-acting elements in the promoter regions of the *ZmDUF966* family in maize. Different colored symbols represent cis-acting elements with distinct functions. The scale bar at the bottom indicates the distance (bp) from the start codon (ATG). The elements are primarily categorized into four groups: light responsiveness, plant growth and development (e.g., G-box, O2-site), phytohormone responsiveness (e.g., ABRE, TCA-element, CGTCA-motif), and abiotic stress responsiveness (e.g., LTR, MBS). The color gradient in the heatmap represents the quantity of elements, with green representing a lower count, red representing a higher count, and blank spaces representing an absence of the element in the respective gene promoter.

To further investigate the regulatory mechanisms of the *ZmDUF966* gene family at the transcriptional level, 2000 bp sequences upstream of the start codon (ATG) for each member were extracted, and *cis*-acting elements were predicted using the PlantCARE database (Figure 4). The analysis revealed that the promoter regions of *ZmDUF966* family members are enriched with numerous regulatory elements associated with abiotic stress response, phytohormone signaling, and plant growth and development. Regarding abiotic stress, elements such as the drought-responsive MBS, the low-temperature-responsive LTR, and TC-rich repeats involved in defense and stress responses were prevalent. In terms of hormone response, a large number of abscisic acid-responsive elements (ABRE), methyl jasmonate-responsive motifs (TGACG/CGTCA-motif), and salicylic acid-responsive elements (TCA-element) were identified. Notably, analysis of the *ZmDUF966-10* promoter showed that it contains multiple ABREs-consistent with the subsequent identification of an ABA-induced protein via yeast two-hybrid screening-as well as prominent LTR (low-temperature responsive) elements. This structural evidence is consistent with the strong cold-inducible expression of *ZmDUF966-10* observed in the RNA-seq data, suggesting putative transcriptional regulation by cold and ABA signals, but experimental validation of these cis-element functions is needed. The distribution patterns of these cis-acting elements suggest that *ZmDUF966* family members serve as critical bridges in the crosstalk between multiple stress signaling pathways in maize.

### 3.5. miRNA Prediction and Regulatory Network Analysis of the ZmDUF966 Gene Family

**Figure 5:**
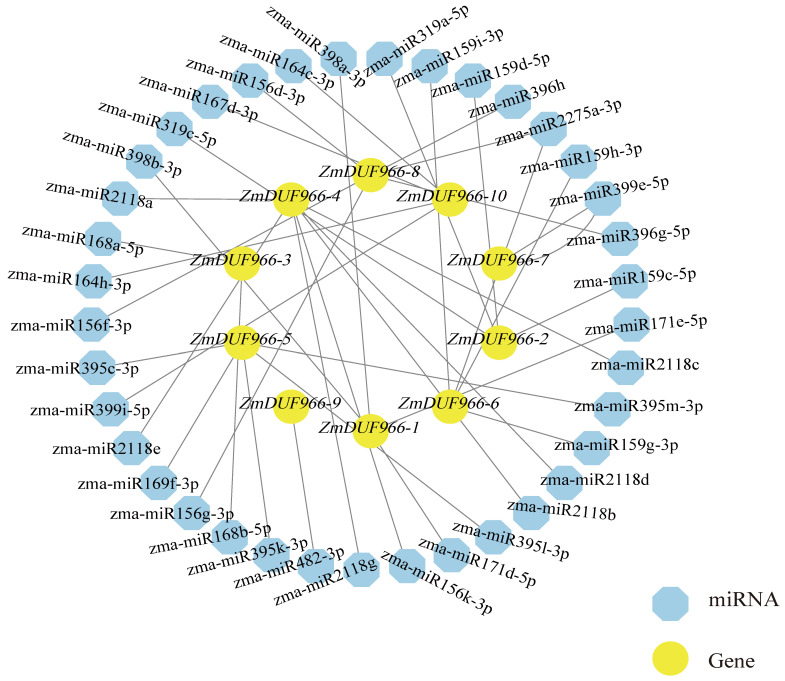
Analysis of the miRNA regulatory network for *ZmDUF966* family members. Blue octagon represent predicted miRNA nodes, while yellow circles denote the targeted *ZmDUF966* gene nodes. The gray lines connecting them indicate the regulatory interactions.

Beyond transcriptional regulation, miRNA-mediated mechanisms represent another critical layer in the precise response of plants to environmental signals [32]. In this study, a miRNA regulatory network targeting the *ZmDUF966* family was predicted and constructed (Figure 5). The results revealed that a total of 37 *zma-miRNA* molecules target the *ZmDUF966* gene family, exhibiting complex many-to-many regulatory characteristics. Among them, the *zma-miR159* family (including five members: c/d/g/h/i) emerged as a central regulatory hub, collectively targeting *ZmDUF966-2*, *ZmDUF966-6*, *ZmDUF966-7*, and *ZmDUF966-10*. Furthermore, *ZmDUF966-10* was identified as being synergistically regulated by the *zma-miR396* family (g/h). Given that *miR159* plays a pivotal role in abscisic acid (ABA) signaling and drought resistance [33], and the *miR396* family is typically involved in modulating the balance between growth and defense under stress [34], these findings suggest that the *ZmDUF966* family may function downstream of relevant stress regulatory axes. Within the network, the *zma-miR156* family (d/f/g) specifically targets *ZmDUF966-8*. These highly conserved miRNAs (e.g., *miR156*, *miR159*, and *miR396*) are extensively involved in plant growth, development, and abiotic stress responses. The discovery of this predicted network suggests that the *ZmDUF966* family may be subject to multi-layered regulation, not only by various cis-acting elements at the transcriptional level but also potentially through miRNA-mediated cleavage or translational inhibition at the post-transcriptional level. These computational predictions will experimental confirmation in the next.

### 3.6. GO Functional Enrichment Analysis of ZmDUF966 Family Members

**Figure 6:**
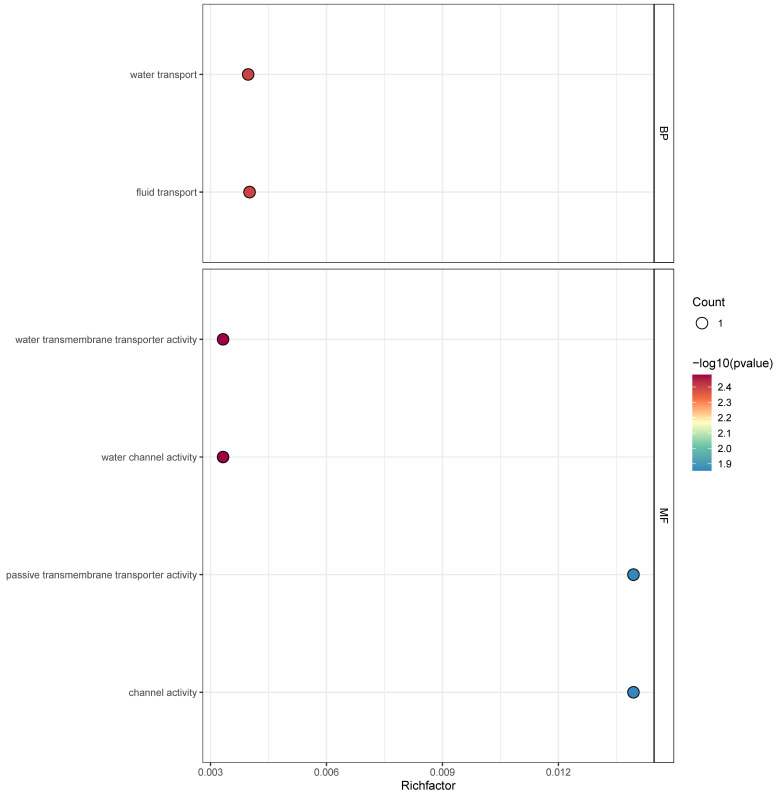
GO functional enrichment analysis of *ZmDUF966* family genes. The biological functions of *ZmDUF966* family members are elucidated across three levels: Biological Process (BP), Cellular Component (CC), and Molecular Function (MF). The horizontal axis represents the Rich factor, while the vertical axis displays the enriched GO terms (Description). The size of the bubbles corresponds to the number of genes enriched in each term (Count), and the color gradient indicates the level of significance, with redder tones representing more significant enrichment.

To predict the potential biological processes involving *ZmDUF966* family members from a functional perspective, a Gene Ontology (GO) enrichment analysis was conducted (Figure 6). The results indicated that the family members are primarily enriched in two major categories: Biological Process (BP) and Molecular Function (MF). Regarding Biological Process, *ZmDUF966* members were significantly enriched in water transport and fluid transport. In terms of Molecular Function, the members were mainly involved in water transmembrane transporter activity, water channel activity, and passive transmembrane transporter activity. These enrichment results possess clear biological implications: *ZmDUF966* family members are highly likely to function by regulating cellular water balance or participating in transmembrane transport under water-related stress conditions.

### 3.7. Transcriptional Response Analysis of ZmDUF966 Family Members to Cold Stress

**Figure 7:**
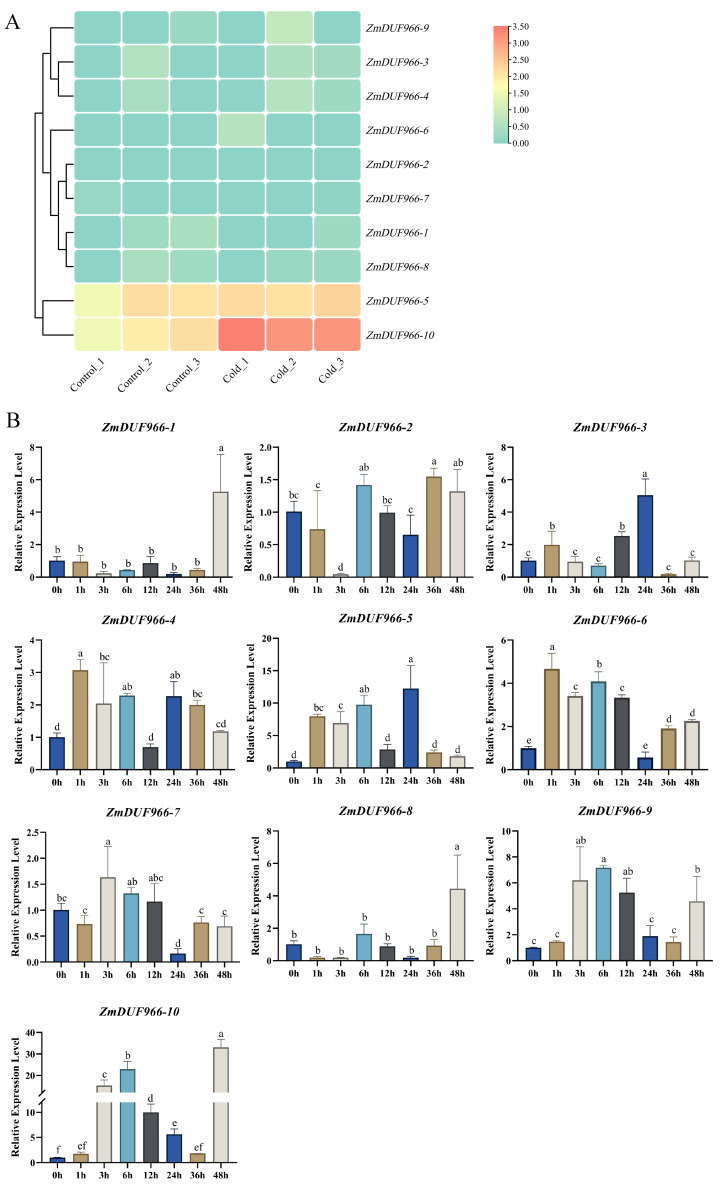
Transcriptional response analysis of *ZmDUF966* family members to cold stress. (**A**) Expression profiling of maize *ZmDUF966* family members under cold stress. The heatmap illustrates the expression changes of 10 members in the control (Control_1-3) and cold-treated (Cold_1-3) groups. The horizontal axis represents different replicates, and the vertical axis represents gene names. The scale bar indicates Log2-transformed expression levels, with red representing up-regulation and blue representing down-regulation. (**B**) Validation of expression patterns for 10 candidate *ZmDUF966* genes under cold stress. Relative expression levels of the 10 *ZmDUF966* family members at different time points (0, 1, 3, 6, 12, 24, 36, and 48 h) of 4 °C cold treatment. The horizontal axis represents treatment time, and the vertical axis represents relative gene expression. Different lowercase letters above the bars indicate statistically significant differences among time points (*p* < 0.05) based on Duncan’s multiple range test. Time points sharing the same letter are not significantly different, while those with different letters differ significantly.

Integrating the transcriptomic data before and after cold stress, *ZmDUF966* family members exhibited highly differentiated response patterns to low-temperature signals. Among the ten members, *ZmDUF966-10* displayed the most prominent positive response. Following cold stress treatment, its expression abundance was drastically up-regulated compared to the control group, manifested as a highly significant deep red in the heatmap, indicating that this gene is a core candidate for the maize response to low-temperature stress. Furthermore, *ZmDUF966-5*, *ZmDUF966-6*, and *ZmDUF966-9* also showed varying degrees of induced expression. In stark contrast, other members such as *ZmDUF966-2* and *ZmDUF966-7* exhibited no obvious response under cold treatment (Figure 7A). These distinct expression behaviors among family members suggest functional specialization of the *ZmDUF966* family during evolution, with *ZmDUF966-10* potentially playing a dominant role in the defense mechanisms against cold injury in maize. This result is highly consistent with the identification of multiple LTR (low-temperature responsive) elements in the preceding promoter analysis, further confirming its biological characteristic of being precisely regulated by cold signals at the transcriptional level.

To further validate the transcriptomic data and elucidate the dynamic response patterns of the *ZmDUF966* gene family to cold stress, RT-qPCR was performed for all ten members. The results showed that the *ZmDUF966* gene family exhibited significantly differentiated expression characteristics during the 0–48 h of 4 °C cold treatment. Most selected genes showed an obvious induction response. Specifically, the expression levels of *ZmDUF966-3*, *-4*, *-5*, *-6*, and *-9* increased with treatment time, all exhibiting a trend of early induction followed by a late decline. The expression of *ZmDUF966-5* showed the most pronounced fluctuations, suggesting these genes may participate in early signal transduction under cold stress. The response of *ZmDUF966-10* was the most intense; it was rapidly induced in the early stage of cold treatment, and although it subsequently declined, it reached a peak at 48 h, with an expression level approximately 35-fold higher than that at 0 h. This indicates that *ZmDUF966-10* may be continuously involved in cold stress signaling. During the initial stages of cold treatment (1–36 h), the expression levels of *ZmDUF966-1* and *ZmDUF966-8* remained generally low, even showing suppression at certain time points. However, at 48 h, both exhibited a sharp surge, reaching approximately 5-fold and 4.5-fold that of the control group, respectively (Figure 7B). These early stability and late burst characteristics suggest their potential involvement in late-stage defense responses to prolonged cold stress. In contrast, *ZmDUF966-2* and *-7* showed almost no up-regulation throughout the treatment. The RT-qPCR results were highly consistent with the transcriptomic heatmap trends, revealing a clear division of labor and temporal relay among *ZmDUF966* members in response to cold stress, with members headed by *ZmDUF966-10* mediating strong cold defense reactions in maize.

### 3.8. Screening and Identification of ZmDUF966-10 Interacting Proteins

**Figure 8:**
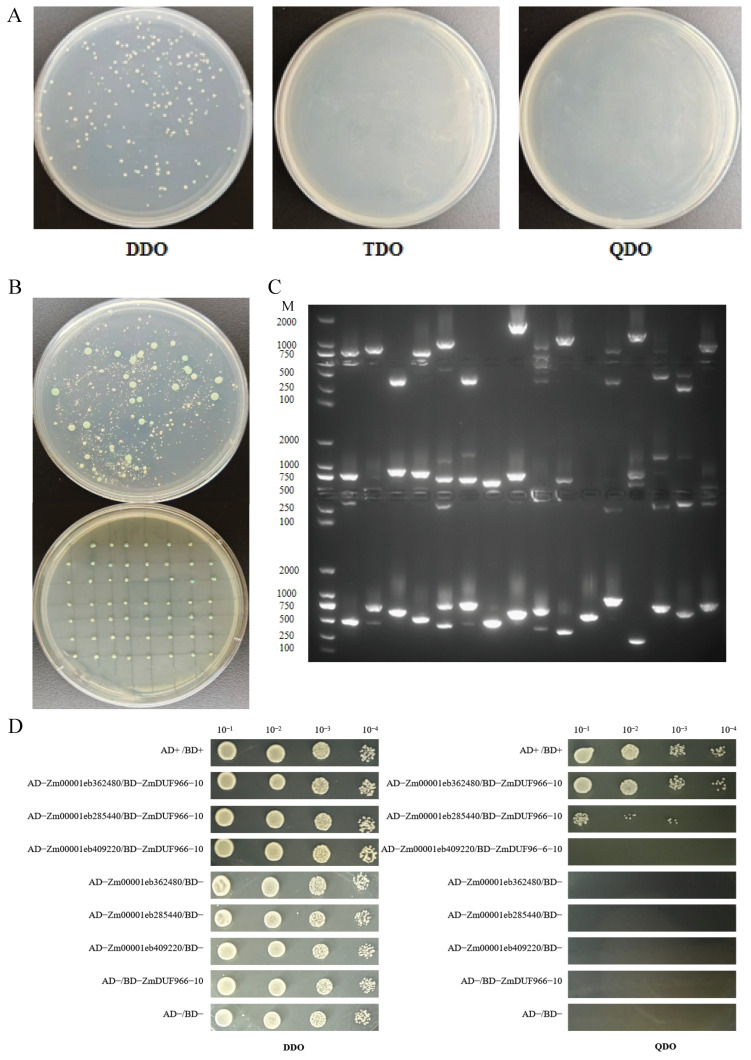
Screening and identification of ZmDUF966-10 interacting proteins. (**A**) Auto-activation assay of pGBKT7-*ZmDUF966-10*. The bait grew on DDO (SD/-Leu/-Trp) medium but failed to grow on TDO (SD/-Trp/-Leu/-His) and QDO (SD/-Trp/-Leu/-His/-Ade) media, showing the lack of auto-activation activity. (**B**) Screening results of co-transformation with the library. Top: primary screening on TDO plates; Bottom: secondary screening of positive clones on QDO/X (SD/-Trp/-Leu/-His/-Ade/X-α-Gal) spot plates. (**C**) PCR detection of 48 positive clones. cDNA inserts were detected via agarose gel electrophoresis, with band sizes ranging from 500 to 1000 bp. M: 100–2000 bp DNA marker. (**D**) Verification of interactions between ZmDUF966-10 and Zm00001eb362480, Zm00001eb285440, and Zm00001eb409220 via Y2H assay. pGBKT7-*ZmDUF966-10* and three prey plasmids were co-transformed into Y2HGold yeast cells. Transformants were spotted onto DDO and QDO media and incubated at 30 °C for 3–5 days. Colony growth on QDO medium indicates a positive interaction. AD+/BD+ co-transformation served as the positive control, while pGADT7 and pGBKT7 (AD-/BD-) co-transformation served as the negative control.

Previous transcriptomic and RT-qPCR results consistently demonstrated that *ZmDUF966-10* strongly responds to abiotic stresses related to low temperature and water deficit. Since plants cope with environmental stresses through complex protein regulatory networks, identifying the interacting partners of ZmDUF966-10 is crucial for elucidating its molecular mechanism. In this study, yeast two-hybrid (Y2H) technology was employed to systematically screen candidate proteins binding to ZmDUF966-10 within a maize nuclear system library. First, auto-activation assays showed that the bait vector pGBKT7-*ZmDUF966-10*, when co-transformed with the pGADT7 empty vector, failed to grow on TDO (SD/-Trp/-Leu/-His) and QDO (SD/-Trp/-Leu/-His/-Ade) deficiency media. This confirmed the absence of auto-activation activity, rendering it suitable for subsequent screening (Figure 8A). During the library screening, over 500 monocultures were obtained, with a calculated total clone count of 1.2 × 10^7^ CFU. This far exceeds the standard for high-capacity library screening (>10^5^ CFU) [35], thereby ensuring sufficient screening coverage (Figure 8A). Approximately 500 primary positive clones were obtained through initial screening on TDO medium. Subsequently, 54 clones were randomly selected for secondary screening via spot assay on QDO/X (SD/-Trp/-Leu/-His/-Ade/X-α-Gal) medium; all selected clones exhibited stable growth activity and blue coloration (Figure 8B). PCR identification of 48 positive clones revealed that the lengths of exogenous inserts were all greater than 250 bp, primarily distributed between 500 and 1000 bp (Figure 8C), which aligns with the distribution standards for library inserts. Sequencing and functional annotation identified several stress-responsive factors among the candidate proteins (Supplementary Data S1), such as an ABA/WDS-induced protein (Zm00001eb362480). The acquisition of these candidate partners provides vital clues for revealing the specific execution mechanisms of ZmDUF966-10 within the maize stress-resistance regulatory network.

Based on functional relevance and fragment integrity, three representative candidate proteins were selected for point-to-point validation to confirm the authenticity and intensity differences in the interactions. These proteins included Zm00001eb362480 (ABA/WDS-induced protein), Zm00001eb285440 (PROTEASE DO-LIKE 9, PDL9), and Zm00001eb409220 (Photosystem I subunit PsaN). Experimental results (Figure 8D) showed that all co-transformed strains exhibited robust growth on double-dropout DDO (SD/-Leu/-Trp) medium, indicating the successful transformation of both bait and prey vectors into the yeast cells. On quadruple-dropout QDO (SD/-Leu/-Trp/-His/-Ade) medium, strains co-transformed with pGADT7-*Zm00001eb362480* and pGBKT7-*ZmDUF966-10* grew vigorously. This result provides preliminary evidence of a physical interaction between ZmDUF966-10 and the ABA/WDS-induced protein in yeast, suggesting a potential role in ABA-mediated stress signaling. Strains co-transformed with pGADT7-Zm00001eb285440 (PDL9) showed very weak growth on QDO medium, indicating a weak interaction. In contrast, strains co-transformed with pGADT7-*Zm00001eb409220 (PsaN)* failed to grow on QDO medium, proving that ZmDUF966-10 does not interact with the chloroplast-localized PsaN. These results strongly substantiate a highly specific and robust interaction between ZmDUF966-10 and the ABA/WDS-induced protein. The identification of this preliminary interacting factor is consistent with the GO-predicted association with water metabolism and provides an important lead for further analyzing the molecular mechanisms of the *ZmDUF966* family in the maize cold-stress signal transduction network.

## 4. Discussion

In this study, ten *ZmDUF966* family members were identified across the maize genome. Phylogenetic and synteny analyses demonstrated significant collinearity and close evolutionary relationships between maize and rice *DUF966* members, suggesting high functional conservation of this family during the evolution of Poaceae. Previous research has indicated that the *DUF966* family (e.g., *OsDSR3* in rice) plays vital roles in plant growth, development, and stress signal transduction [17]. Our findings show high consistency in motif composition among *ZmDUF966* members, suggesting they execute similar biological functions, such as nucleic acid binding or mediating specific protein–protein interactions, through these conserved domains to coordinately regulate environmental adaptation in maize. While systematic characterization of *DUF966* gene families has been reported in rice [17], wheat and tomato [18], the present study represents shows that this study is the first genome-wide identification and functional analysis of the *DUF966* family specifically in maize. The discovery of *ZmDUF966-10* as a cold-responsive candidate gene, together with its preliminary interaction with an ABA/WDS protein, provides species-specific insights with relevance to maize cold stress biology.

Transcriptomic sequencing and RT-qPCR validation revealed that *ZmDUF966* family members are extensively involved in the response to cold stress. Notably, the expression of *ZmDUF966-10* exhibited dramatic fluctuations under low-temperature treatment, with a maximum induction of approximately 35-fold at 48 h Such a potent and sustained induction pattern is consistent with a potential role in cold stress signaling; however, functional genetic validation through overexpression or loss-of-function studies is needed to confirm its regulatory significance. Furthermore, promoter analysis revealed an enrichment of LTR (low-temperature responsive) elements [36], ABREs (abscisic acid-responsive elements) [37], and various multiple functions MYB/MYC transcription factors binding sites [38]. This provides a molecular basis for the high sensitivity of *ZmDUF966* members to cold and related secondary stresses, such as osmotic stress. As a core respondent to cold stress, the high-level expression of *ZmDUF966-10* likely represents a vital defense strategy for maize in low-temperature environments. Notably, while *ZmDUF966-10* showed an initial early induction, its expression reached a maximum at 48 h of cold treatment. Such a temporal expression pattern may indicate that *ZmDUF966-10* participates in sustained cold adaptation processes, contributing to membrane stabilization or osmotic adjustment during prolonged cold exposure.

At the post-transcriptional level, miRNA-mediated mechanisms are crucial for the precise response of plants to environmental cues [39]. miRNAs are a class of endogenous non-coding RNAs that regulate gene expression in eukaryotes by recognizing target mRNAs through complementary base pairing. This binding guides the silencing complex to degrade the target mRNA or inhibit its translation. Consequently, the spatiotemporal expression patterns of target genes are coordinated with transcriptional regulation and guided by transcription factors [40]. Our predicted regulatory network suggests that the *ZmDUF966* family may be regulated by several evolutionarily conserved miRNAs, including *zma-miR159*, *zma-miR156*, and *zma-miR396*. Specifically, *zma-miR159* is predicted to target *ZmDUF966-10*. It is well-established that *miR159* participates in ABA signaling and stress responses by regulating transcription factors such as MYBs [41]; thus, the putative regulation of *ZmDUF966-10* by *miR159* suggests a potential association with ABA-dependent stress pathways. Additionally, *miR396* is a conserved miRNA in both dicots and monocots; in *Arabidopsis*, *miR396* targets six *Growth-Regulating Factor (GRF)* genes to modulate leaf growth [42,43]. Our finding that *zma-miR396* coordinately regulates *ZmDUF966* members suggests it may mediate the dynamic balance between growth and defense under cold stress. This multi-layered “miRNA-mRNA” regulatory network ensures fine-tuned control of *ZmDUF966* expression in complex natural environments.

Bioinformatic analysis identified *ZmDUF966-10* as a potential core member of the family, and subsequent yeast two-hybrid experiments confirmed its specific physical interaction with an ABA/WDS-induced protein (Zm00001eb362480). ABA/WDS proteins, also known as ASR (Abscisic acid-, Stress-, and Ripening-induced) proteins, are highly hydrophilic, tissue-specific DNA-binding proteins [44,45]. The ASR gene family has been reported to exist across gymnosperms, monocots, and dicots [46]. It serves as a key component in multiple regulatory networks [47], participating in plant development, senescence, and fruit ripening, and plays a crucial role in the tolerance of most plants to abiotic stresses such as drought, cold, and salt stress, including *OsASR1* [48], *ZmASR1* [49], and *TaASR1* [50], often under ABA regulation [51]. Our GO enrichment analysis predicted that *ZmDUF966* members may be associated with water transport and water channel activity. Since cold stress often causes root water-uptake inhibition and induces physiological drought [52], we speculate that the preliminary interaction observed between ZmDUF966-10 and the ABA/WDS protein may relate to osmotic adjustment during water stress; however, this proposed mechanism requires validation through in planta functional studies, including subcellular co-localization, BiFC, and genetic complementation experiments.

However, several limitations of the present study should be acknowledged. First, the functional characterization of *ZmDUF966-10* in this work is based primarily on transcriptomic, computational, and preliminary yeast two-hybrid data; no functional genetic validation (e.g., overexpression, RNAi silencing, or CRISPR-based knockout) has been performed. Therefore, *ZmDUF966-10* can currently serve as a candidate gene for the corresponding cold stress response. Secondly, due to the limited number of members in the *ZmDUF966* gene family, the GO enrichment analysis results should be regarded as preliminary predictions. Third, the protein–protein interaction identified by Y2H has not been confirmed by independent in planta methods such as BiFC or co-immunoprecipitation. Future studies should focus on: generating transgenic plants with altered *ZmDUF966-10* expression to assess cold-stress phenotypes; confirming the protein interaction in planta; and elucidating the precise molecular mechanism underlying *ZmDUF966*-*10* mediated cold tolerance.

In summary, *ZmDUF966-10* is a key component of the maize cold-response signaling network, achieving multi-dimensional synergistic regulation by integrating post-transcriptional miRNA control and physical protein–protein interaction with an ABA/WDS protein. These findings provide new perspectives on the molecular mechanisms of cold resistance in maize and offer a promising candidate gene for the molecular breeding of cold-tolerant maize varieties.

## 5. Conclusions

In conclusion, this study systematically identified the *ZmDUF966* gene family in maize, revealing high conservation in terms of phylogenetic relationships, gene structures, and conserved motifs. Integrated analyses of cis-acting elements, miRNA regulatory networks, and GO functional enrichment clarified the potential roles of *ZmDUF966* members in regulating water metabolism and responding to low-temperature signals. RT-qPCR data demonstrated that the core member, *ZmDUF966-10*, is significantly induced by and strongly responds to cold stress. Furthermore, yeast two-hybrid assays validated the physical interaction between ZmDUF966-10 and an ABA/WDS-induced protein. These results suggest that *ZmDUF966-10* is a promising candidate gene involved in the maize response to cold stress. Its preliminary interaction with the ABA/WDS protein indicates a potential role in cooperating to resist physiological dehydration damage caused by cold injury. This discovery provides a valuable candidate gene resource and theoretical foundation for further functional studies, and may ultimately contribute to the development of cold-tolerant maize germplasm through genetic improvement approaches once functional validation is completed.

## Data Availability

All datasets presented in this study are included in the article/Supplementary Material.

## Supplementary Materials

The following supporting information can be downloaded at: https://www.mdpi.com/article/10.3390/biology15060514/s1, Table S1: Oligonucleotide primers used for RT-qPCR analysis. Table S2: Physicochemical properties of *ZmDUF966* gene family members. Table S3: Ka, Ks and Ka/Ks ratios for duplicated *ZmDUF966* gene pairs in *Zea mays*. Data S1: Information of Yeast Library Screening and Identified Interacting Partners for ZmDUF966-10.
