# Supplementary material for: Identification of the ZmDUF966 Gene Family in Maize, Analysis of Its Expression Under Cold Stress, and Preliminary Investigation of the ZmDUF966-10 Regulatory Network
Source: Biology (Basel). 2026 Mar 23;15(6):514. doi: 10.3390/biology15060514 (PMC13023492; doi:10.3390/biology15060514)
Supplement: Supplementary file 1 [file biology-15-00514-s001.zip › Supplementary Material.pdf]

Table S1. Oligonucleotide primers used for RT-qPCR analysis.

**Name**	**Forward primer (5'→3')**	**Reverse primer (5'→ 3')**
ZmDUF966-1	GTACGTGTGGAACGACCTGT	GCTGGAACCGATCGGAAGAA
ZmDUF966-2	TACATCCTCAAGGGCTCCGA	GGTGCTTGAGGTTCTCCTCC
ZmDUF966-3	CGCGAAGAGGATAAAGCCGA	ACGACAGCCTCGATCTTGAC
ZmDUF966-4	GCCAAACGCCGAAAAGAACT	TGCTCCTCCTCCTGCTTTTG
ZmDUF966-5	TCCTTCTGGAGCTCGGGTTA	ACTCCAAAAGAGTGATTCATTGC
ZmDUF966-6	TGAACTCTGTCAGTGCTCGG	ATGATCTCCCTGCAGCCATC
ZmDUF966-7	CTCAAGGTGGATCCCCACAC	GGACCCTTTGAGGTTCTCGG
ZmDUF966-8	AGCAACACACAAGTGGGACA	TTTTCTGCCCCACACAGCTT
ZmDUF966-9	CGGTCATCCTCCTGCAACG	GGCCACCGTGGATTTAGTCA
ZmDUF966-10	TACAACGCGGAAAGACGACA	GAGATGACCAGGTATCCGTCG
ZmActin	TACGAGATGCCTGATGGTCAGGTC	TGGAGTTGTACGTGGCCTCATGGA

Table S2. Physicochemical properties of ZmDUF966 gene family members

**Gene_ID**	**Name**	**Protein Length**	**CDS Length (nt)**	**Molecular Weight (Da)**	**Isoelectric Point (pI)**	**Hydrophobicity (GRAVY)**	**Localizations**
Zm00001eb008380_P001	ZmDUF966-1	414	1242	44450.09	8.94	-0.79	Nucleus
Zm00001eb039020_P001	ZmDUF966-2	594	1782	63971.39	8.15	-0.79	Cytoplasm
Zm00001eb053610_P001	ZmDUF966-3	555	1665	62234.42	9.49	-0.84	Cytoplasm
Zm00001eb151570_P001	ZmDUF966-4	415	1245	46246.84	9.13	-1.17	Nucleus
Zm00001eb175850_P001	ZmDUF966-5	625	1875	67968.2	8.34	-0.65	Cytoplasm
Zm00001eb217090_P001	ZmDUF966-6	635	1905	70253.23	7.24	-0.84	Cytoplasm
Zm00001eb253300_P001	ZmDUF966-7	322	966	34527.83	8.19	0.35	Endoplasmic reticulum
Zm00001eb307740_P001	ZmDUF966-8	595	1785	65403.32	8.64	-0.61	Cytoplasm
Zm00001eb350150_P001	ZmDUF966-9	386	1158	41804.06	8.75	-0.71	Nucleus
Zm00001eb368040_P001	ZmDUF966-10	396	1188	43205.1	9.78	-0.66	Cytoplasm

Table S3. Ka, Ks and Ka/Ks ratios for duplicated *ZmDUF966* gene pairs in *Zea mays*.

**Seq_1**	**Seq_2**	**Ka**	**Ks**	**Ka_Ks**
ZmDUF966-2	ZmDUF966-5	0.07431305908383197	0.49386301066833793	0.15047302081454764
ZmDUF966-2	ZmDUF966-3	0.5189518450302356	1.4200395101784467	0.3654488775210364
ZmDUF966-2	ZmDUF966-6	0.581868056087457	1.4054383087793911	0.4140118085957138
ZmDUF966-2	ZmDUF966-7	0.44518657705498693	1.4392192323915929	0.30932506114110725
ZmDUF966-3	ZmDUF966-6	0.13163200101665593	0.4026356426800888	0.32692585321176637
ZmDUF966-3	ZmDUF966-7	0.34785136956515655	0.7806510972877718	0.44559134134788503
ZmDUF966-6	ZmDUF966-7	0.3267826650237977	0.8629492843973771	0.37868119359065194
